# The Foreign Journals: Chemistry

**Published:** 1841-07

**Authors:** 


### ANIMAL CHEMISTRY.

*On the Chemical Analysis of the Blood in its morbid conditions.*
By Dr. Louis Mandl.

The object of this series of papers is to point out the defects and fallacies of the present modes of analyzing the blood, and especially of estimating the *quantities* of its several principal constituents.

1. Three methods have been most commonly employed to separate the fibrine, but all are fallacious. The first consists in squeezing the clot of blood in linen, so as to get rid of its serum, and then repeatedly washing the remainder. Now the *clot* of blood has by no means always the same characters; some clots are hard and dense, and can only with difficulty be broken up, and these may certainly have all their fibrine, which forms compact stringy masses, retained within the linen. But others are soft and friable, and when these are broken up and squeezed numberless particles of fibrine pass through the fine meshes of the linen, and what remains is but a small portion of what really existed in the blood. Independently therefore of the varying effects of more or less force in pressing the clot, and of the coarseness or fineness of the linen used, it is impossible that by this method, (which was generally employed by M. Denis,) any just idea should be formed of the respective quantities of fibrine in coagula whose physical qualities are different; for in all soft coagula, the fibrine breaks up into pieces small enough to pass through the linen with the serum and colouring matter.

The second method, by stirring the blood, is not less defective. Besides that, even under the best of circumstances, it is not so easy to stir the blood as to get all its fibrine in shreds, here again as in the preceding case the fibrine may be in such a state as to render its separation impossible. In blood mixed with pus for example, it forms in coagulating particles which are either so small that they can hardly be seen, or pieces which stick to the walls of the vessel, or swim in the rest of the blood, and cannot possibly be separated from it. And in many other circumstances a similar condition exists in a less degree, so that this method can be employed only in cases in which the fibrine is disposed to coagulate firmly and in large masses, and has not its tendency to become solid, interfered with by the presence of purulent, or saline, or other substances in excess in the blood.

The method of Berzelius, that of compressing slices of the coagulum in blotting paper, and when they have ceased to give out moisture washing them till they are colourless, is open to different but almost equal objections. In washing the remains of the coagulum one of two things will happen, either the blood-globules that are retained in it will be washed out entire, and then they will carry with them small pieces of the fibrine itself, or else their fluid contents and their colouring matter only will be removed, and their own membranes and those of their nuclei (which cannot be chemically distinguished from fibrine) will remain. In the former case the quantity of fibrine will appear less than it really was; in the latter it will seem more, perhaps much more; nor can any dexterity of manipulation avoid these errors. Besides from one kind of clot, (the firm one of inflammatory blood), it is almost impossible to wash away all the globules without breaking it up; and from others (the softer kinds formed in typhoid fever, &c.) the fibrine separates sometimes in particles almost as small as the globules themselves, and cannot be retained on a filter; numberless particles of it may be detected with the microscope in each portion of water that passes through in the process of washing, and many more when (as is commonly the case) the fibrine is washed, not on a filter, but in a vessel of water.

In whatever method it be obtained, the fibrine is subsequently macerated for a considerable time in water, in which, if the process be contained for twelve or more hours, as it commonly is, decomposition takes place, the water becoming turbid, and the fibrine gelatinous. And after this it has to be dried, an operation requiring great delicacy and judgment to ensure that at the end of it water shall not be still retained in the organic substance which so readily absorbs it.

And after all it is very doubtful whether under the best of circumstances the fibrine extracted from the blood is all that it really contained, for none of the analyses yet published take into account that which in all probability exists in the envelopes and contents of the globules, and which may well amount to more than there is in the *liquor sanguinis*. For several things are quite inexplicable on the supposition that as commonly stated there are only three parts of fibrine in 1000 of blood; as for example, the immense quantities effused in false membranes, amounting in some cases to more than the whole mass of blood is said to contain, and many others.

If it be said that in spite of all these sources of fallacy the analyses of different chemists closely accord ; reference need only be made to those analyses, and it will appear that the quantities of fibrine said to exist vary from 0·75 (Berzelius) to 4·96 (Müller) parts in 1000. Besides, these analyses were made on healthy specimens of blood ; the difficulties are greatly increased when it is in a morbid state, when the determination of its quantity of fibrine is most important.

2. The composition of the globules is as yet rather uncertain, but the author has little doubt that their contents are in part fibrine, by the coagulation of which the nucleus is formed ; and he thinks that their membranes are also fibrine, coloured with hematosine. Now, as already stated, this fibrine within the globules has not in any analysis hitherto published been taken notice of ; nor is it right to estimate its quantity from that of the albumen (as it has been supposed to be) which chemists have obtained from the globules : for it is difficult in any case to collect all the globules in a sample of blood ; a number of them always float in the serum, and the more the softer the clot is, so that in many cases the serum itself is deep red ; and moreover, in the ordinary plans of treatment, the clot is never obtained completely free from serum, and therefore from a greater quantity of albuminous matter than properly belongs to its globules. In short there is not at present any method known by which the globules can be all removed from a portion of blood, and obtained separately from its serum and other principles ; and there cannot therefore be any certainty in the calculations of their weight or of the quantity of fibrine or albumen which they contain.

3. The fallacies in the analysis of the serum depend chiefly on the quantity of globules and of portions of fibrine that may be suspended in it. These in the healthy state of the blood are unimportant, but in its diseased states they may give rise to considerable errors.

In the next division of his subject the author treats of the coagulation of the blood, and the formation of the buffy coat, of the chemical nature of which, he rightly says, nothing is at present known. [But under this head we find nothing worthy of being abstracted.]

In the application of his objections, M. Mandl takes two classes of cases, namely, those in which the blood is said to be poor in fibrine, including scurvy, putrid fevers, typhoid fevers, &c. and those in which it is described as rich in globules. For the first the quantity of fibrine will appear small in all cases in which a firm clot does not form, that is, not only when there is really little fibrine, but when that which does exist, after coagulating, does not contract and form a dense hard clot. And this may depend on the presence of pus or other morbid fluids in the blood, or, still more probably, on an excess of alkaline salts. The latter are known to exist in excess in scurvy, and perhaps may be found so in the other diseases in which the blood has been less carefully analysed. At any rate, it is certain that we have at present no means of ascertaining the real quantity of fibrine in a soft clot, and therefore no right to assume a deficiency of it in any cases till the softness of the clot is proved not to depend on some other cause than that deficiency. Similar difficulties attend the appreciation of the true quantity of globules ; and hence the diversities of the statements of authors respecting their increase or decrease in different diseases.

The last part of these papers contains general reflections on the application of the chemical researches on the blood to pathology. [But in this also the remarks are trivial. Indeed, the author has evidently much more skill in finding fault than in improving defects. We have ourselves long thought that in the matter of the blood, physiologists have too implicitly received the statements of chemists ; and we hope that the brief abstract we have given of M. Mandl's papers, though they do no more than point out the numerous and unavoidable fallacies of these analyses, will engender caution in drawing conclusions from such evidently insecure premises.]

*Archives Générales de Médecine. Decemb.* 1840, *and Jan. and Fevr.* 1841.

